# Parallel evolution of behaviour during independent host‐shifts following maize introduction into Asia and Europe

**DOI:** 10.1111/eva.12481

**Published:** 2017-06-17

**Authors:** Vincent Calcagno, Clémentine Mitoyen, Philippe Audiot, Sergine Ponsard, Gui‐Zhen Gao, Zhao‐Zhi Lu, Zhen‐Ying Wang, Kang‐Lai He, Denis Bourguet

**Affiliations:** ^1^ INRA Université Nice Côte d'Azur CNRS, ISA Sophia Antipolis France; ^2^ INRA UMR Centre de Biologie pour la Gestion des Populations (CBGP) Montferrier‐sur‐Lez France; ^3^ UMR5174 EDB (Laboratoire Évolution & Diversité Biologique) CNRS ENFA Université Toulouse 3 Paul Sabatier Toulouse France; ^4^ CNRS UMR5174 EDB Toulouse France; ^5^ Xinjiang Institute of Ecology and Geography Chinese Academy of Sciences Urumqi China; ^6^ College of Forestry and Horticulture Xinjiang Agricultural University Urumqi China; ^7^ State Key Laboratory for the Biology of the Plant Diseases and Insect Pests Institute of Plant Protection Chinese Academy of Agricultural Sciences Beijing China

**Keywords:** agricultural pests, behavioural adaptation, geotaxis, harvest selection, host‐shift, *Ostrinia* spp, parallel evolution

## Abstract

Maize was introduced into opposite sides of Eurasia 500 years ago, in Western Europe and in Asia. This caused two host‐shifts in the phytophagous genus *Ostrinia*;* O. nubilalis* (the European corn borer; ECB) and *O. furnacalis* (the Asian corn borer; ACB) are now major pests of maize worldwide. They originated independently from Dicot‐feeding ancestors, similar to *O. scapulalis* (the Adzuki bean borer; ABB). Unlike other host‐plants, maize is yearly harvested, and harvesting practices impose severe mortality on larvae found above the cut‐off line. Positive geotaxis in the ECB has been proposed as a behavioural adaptation to harvesting practices, allowing larvae to move below the cut‐off line and thus escape harvest mortality. Here, we test whether the same behavioural adaptation evolved independently in Europe and in Asia. We sampled eight genetically differentiated ECB, ACB and ABB populations in France and China and monitored geotaxis through the entire larval development in artificial stacks mimicking maize stems. We find that all ECB and ACB populations show a similar tendency to move down during the latest larval stages, a behaviour not observed in any European or Asian ABB population. The behaviour is robustly expressed regardless of larval density, development mode or environmental conditions. Our results indicate that maize introduction triggered parallel behavioural adaptations in Europe and Asia, harvest selection presumably being the main driver.

## 1 INTRODUCTION

Anthropogenic selection pressures can elicit fast adaptive responses, sometimes occurring over as few as a dozen generations (Palumbi, 2001). Life‐history or morphological evolution in response to anthropogenic selection has been observed in several plant, fish and mammal taxa (Allendorf & Hard, 2009; Biro & Sampson, 2015). Behaviour can also evolve as a resistance mechanism to man's impact in the wild, although there are comparatively few examples. For instance, aphids *Myzus persicae* developed the capacity to discriminate fields treated with neonicotinoïds and adapt dispersal behaviour accordingly (Fray et al., 2014), and *Diabrotica virgifera virgifera* populations with reduced host fidelity have emerged following the implementation of corn‐soya bean rotations as a pest‐control strategy (Knolhoff, Onstad, Spencer, & Levine, 2006).

**Figure 1:**
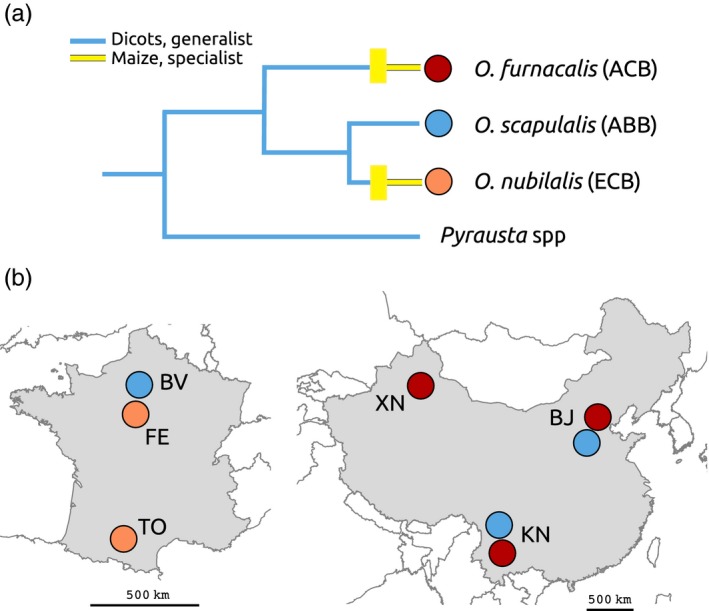
(a) Most likely evolutionary history of the three *Ostrinia* species studied (adapted from Ishikawa et al., 1999). Yellow rectangles indicate host‐shifts to maize. Branch lengths are arbitrary, and the most closely related genus *Pyrausta* is shown as an out‐group (Regier et al., 2012). (b) Sampling localities in France and in China. Note that two species were sometimes sampled in the same locality

Here, we investigate an original example of man‐induced behavioural evolution in the genus *Ostrinia* (Lepidoptera: Crambidae). Phytophagous insects in this genus are essentially found in Asia and Europe, but also in North America, North Africa and Oceania (Frolov, Bourguet, & Ponsard, 2007; Hudon, LeRoux, & Harcourt, 1989). The European corn borer (ECB; *O. nubilalis* Hübner) specialized on maize (Bethenod et al., 2005; Calcagno, Thomas, & Bourguet, 2007; Goto & Funayama, 1994; Leniaud et al., 2006; Malausa et al., 2008; Orsucci et al., 2016). It probably originated in Europe but subsequently spread to Africa and, notoriously, North America, where it was accidentally introduced in the early 19th century (Caffrey & Worthley, 1927). The ECB is very closely related to *O. scapulalis* Walker (the Adzuki bean borer, ABB; see Figure 1), the two taxa being regarded as host races until recently (Malausa et al., 2005). The ABB is found throughout Eurasia. Although morphologically indistinguishable from the ECB (Frolov et al., 2007), the ABB feeds on Dicots, mainly mugwort (*Artemisia vulgaris*) and occasionally hop (*Humulus lupulus*), hemp (*Cannabis sativa*) and other species, but not on maize, as is the ancestral state in this group (Figure 1). The ECB has become an important agricultural pest in Europe and North America. Females deposit eggmasses on the underside of maize leaves, and after initially feeding on leaves, larvae burrow into stalks, where they spend the rest of their lifetime—3–6 weeks—tunnelling and destroying vascular tissues and damaging ears. Fifth instars either pupate or enter diapause, depending on genetic and environmental factors. One generation per year is observed in northern areas, but in regions with favourable climatic conditions, several generations can occur. In autumn, low temperature and declining day‐length trigger diapause. Diapausing larvae remain motionless, concealed inside the stem until spring.

Importantly, man has continuously harvested maize during the last 500 years. Harvesting techniques have varied over time, but always involved cutting‐off the upper part of the stalks, which were subsequently burnt, destroyed, fed to cattle or used for various other purposes. As corn borers diapause inside maize stalks at the time of harvest, larvae in the upper part of the stalks suffer high mortality. Destroying maize stalks after harvesting was actually encouraged as a way to control the pest (Caffrey & Worthley, 1927). This situation has culminated in the last 100 years or so, following the wide adoption of mechanical harvesting: combined harvesters kill virtually all larvae above the cut‐off line (Calcagno, Bonhomme, Thomas, Singer, & Bourguet, 2010; Chen, Song, Wang, & Wang, 2016). This dramatic vertical gradient in survival should exert a strong selection pressure for larvae to move closer to the ground prior to harvesting, which typically occurs at the end of larval development, just before the onset of diapause. In contrast, as the ABB's hosts are not cut or grazed by large herbivores, survival rate is not expected to show such vertical variation. Comparing the vertical movements of ECB and European ABB, Calcagno et al. (2010) found that ECB exhibited positive geotaxis (locomotion towards the ground; Toma, White, Hirsch, & Greenspan, 2002; Bourguet, Gair, Mattice, & Whitlock, 2003) in late instars, whereas ABB larvae showed no such behaviour. This behavioural difference translated into contrasted vertical distributions of larvae in the field at the time of harvest. The harvest‐selection intensity favouring the ECBs over the ABBs on maize was estimated in the range 40%–70% (Calcagno et al., 2010). The authors consequently hypothesized that geotaxis of the last larval stage evolved as an adaptation to maize harvesting.

However, observing a difference between a single pair of species offers little evidence that the difference actually evolved as an adaptation, or that the process would be predictable. Fortunately, the ECB is not the only *Ostrinia* species that shifted to maize. *O. furnacalis* Guenée (the Asian corn borer; ACB) is also specialized on maize and is a major agricultural pest in Asia (Alpuerto, 1985; Goto & Funayama, 1994; Hattori & Mutuura, 1987; Ishikawa et al., 1999; Lu, Yang, & Hu, 1996; Talekar et al., 1991). The ACB and the ECB are allopatric, with a very narrow range overlap in northwestern China (Bourguet et al., 2014; Li & Gao, 1985; Wang, Yan, Li, & Li, 1995; Xu, Sun, Wang, & Li, 1998). Like the ECB, the ACB is difficult to distinguish morphologically from the ABB, with which it is parapatric in Asia (Mutuura & Munroe, 1970). The two corn borers are quite different genetically, the ECB being closer to the ABB than to the ACB in the *Ostrinia* genus (Figure 1a; Ishikawa et al., 1999), and genome‐scan approaches suggest different genetic architectures of maize adaptation in the European and Asian species (Alexandre et al., 2013). ACB and ECB thus constitute an interesting example of parallel host‐shifts from Dicots to maize that occurred independently some 500 years ago, following the nearly simultaneous introductions of the crop into Western Europe and Asia (Gay, 1999). When faced with similar selective pressures, species are expected to evolve similar characters, resulting in a so‐called parallel or repeated evolution (Arendt & Reznick, 2008; Schluter & Nagel, 1995). These situations offer powerful opportunities to test the repeatability of host‐shift‐associated evolutionary changes and infer their adaptive nature (Nosil, Crespi, & Sandoval, 2002).

In this study, we test whether the two *Ostrinia* species that have shifted to maize, the ECB in Europe and the ACB in Asia, underwent parallel evolution of the same positive geotaxis behaviour. We simultaneously monitored, in controlled conditions, the geotactic preferences of the three species involved, ABB, ACB and ECB, over their entire larval development. We compared several populations from each species to assess intraspecific variation in geotactic behaviour. We further tested whether the expression of the geotaxis phenotype depended on the developmental pathway taken by larvae (diapause vs. direct development).

## 2 MATERIALS AND METHODS

### 2.1 Sampling and rearing

During autumn and winter 2014, eight populations of diapausing *Ostrinia* larvae were sampled in France and China (Figure 1b). Three ABB populations were sampled, in Beauvais (BV, France, 45°25′N, 2°03′E), Beijing (BJ, China, 40°03′N, 116°08′E) and Kunming (KN, China, 25°02′N, 102°43′E). Two ECB populations were sampled, in Toulouse (TO, France, 43°23′N, 1°43′E) and Feucherolles (FE, France, 48°52′N, 1°58′E). Finally, three ACB populations were sampled, in Beijing, Kunming and Xinjiang (XN, China, 43°49′N, 87°37′E). Each population was named by location followed by species name—*for example* BJ ACB, BJ ABB, TO ECB and so forth. For each population, 300–800 larvae were hand‐collected in the field. ACB and ECB larvae were collected on maize, whereas ABB larvae were collected on mugwort or hop. Larvae were kept at 5°C to sustain diapause. They are referred to as generation 0 (G0).

In December 2014, 200–500 G0 larvae from each of the eight populations were placed under diapause‐breaking conditions (L:D 25:23°C/16:8 hr, with moist cotton pads). Adult moths emerged ca. 3 weeks later. About one hundred adults per population were thus obtained and placed in mating cages for reproduction. Resulting eggmasses were used to initiate the first laboratory‐reared generation (G1). G1 larvae were reared on a standard artificial diet (Gahukar, 1975) at 25°C ± 2°C under a L:D 16:8 hr until they became adults. G1 adults were caged for mating and produced G2 eggmasses. G2 individuals were used for experiments, to avoid potential maternal effects.

### 2.2 Geotaxis assays

G2 larvae were grown in artificial maize stalks, consisting of seven stacked cylindrical plastic vials (60 mm diam. × 45 mm height) connected by six holes (5 mm diam. each) drilled through the lids/bottoms to permit vertical movements of larvae. This is a slight simplification of Calcagno et al.'s (2010) protocol, our stacks containing seven instead of 11 vials, allowing to monitor more populations while maintaining a similar number of replicates per population.

The vertical distribution of larvae in the stacks was monitored through their entire development (12 weeks) from first instar to either diapausing larva or pupa. We initially introduced 40 randomly sampled G2 first instars into the central vial of each stack. Each vial was filled with a standardized nonlimiting amount of diet. Diet was renewed every week during the first 6 weeks and every 2 weeks for the rest of the experiment. Stacks were randomly located in a climatic chamber, in complete darkness to prevent any taxis caused by light. To simulate natural conditions and induce diapause, temperature and thermoperiod inside the chambers were progressively decreased from 25:23°C/16:8 hr during the first week, to 16:13°C/12:12 hr during the fifth week and until the end of the experiment. We created 20 stacks (replicates) for each of the eight populations. Every week for the first 7 weeks, and then on the 10th and the 12th weeks, we recorded the number of G2 larvae and/or pupae found in each vial of each stack. Dead individuals were removed without being counted. After counting, surviving larvae were transferred vial‐to‐vial into a newly assembled stack, with fresh diet, thus keeping their vertical positions unchanged. After 6 weeks, we no longer systematically renewed the stacks, as larvae had mostly stopped feeding. After 12 weeks, larvae no longer moved or fed and had either turned into pupae or entered diapause, adopting the typical “question‐mark” posture of diapausing larvae in the field. Thus, the experiment was stopped.

For each of two populations (BJ ACB and TO ECB), we created 20 additional stacks. These stacks were kept at 25:23°C/16:8 hr during the entire duration of the experiment. In populations with facultative diapause (usually, all but the northernmost populations), these conditions cause fifth instars to develop directly into pupae. As temperature speeds up development, experiments were shorter in this treatment (seven instead of 12 weeks).

### 2.3 Genetic characterization

At the end of the experiments, 40 individuals per population were genotyped at the microsatellite locus *OnT2* (Kim, Coates, Hellmich, Sumerford, & Sappington, 2008). Genotypes were compared to earlier studies (Bourguet et al., 2014) to confirm species identities. *F*_ST_ values were computed between our G2 populations and those from earlier studies, and between our G2 populations, to evaluate the level of genetic differentiation and ascertain that populations had not been accidentally mixed over the experiments.

### 2.4 Data analysis

Vertical distributions of individuals in stacks were modelled using a multinomial distribution, describing the probability that a larva is found in the bottom vial (position −3), the second vial (position −2) and so on up to the top vial (position 3). This represents six free parameters that were estimated by maximum likelihood. To compare the final vertical distributions of our eight populations, we considered all possible models corresponding to all possible groupings of the populations (4,140 different groupings, from all populations being different to all being identical). Models have 6*ng estimated parameters, where ng is the number of different groups (clusters). AIC was computed for each, as well as their relative support, normalized between 0 and 1 (Claeskens & Hjort, 2008). Finally, for each pair of populations, we computed the model‐averaged support for their being identical, by summing the relative weights of all models in which the two populations were grouped. In this approach, we did not provide information on which populations belonged to different species, but rather sought to determine whether the information contained in the vertical distributions was sufficient to recover the number of different species and assign populations to the correct species. To represent differences between populations, we created a graph with populations as nodes and edge‐weights equal to the relative support for grouping. We visualized this graph using a standard attraction‐based layout (Gephi software, version 0.9).

For every population (including in the direct‐development treatments), we determined whether the final vertical distribution was significantly nonsymmetrical around the central vial (position 0). To do this, we tested whether a full description of the vertical distributions (six parameters) was significantly better than a symmetrical distribution (three parameters) using a G‐test with three degrees of freedom. This uses the same multinomial likelihood framework as the clustering analysis above.

We then analysed more specifically the average vertical position of individuals within stacks. For each stack, we computed the average height of individuals as i*n(*i*)/*N* where *i* = −3, −2,…, 3 is the position, from bottom to top, *n*(*i*) is the number of individuals at position *i*, and *N* is the total number of individuals in the stack. This was used as the dependent variable in generalized linear models incorporating population or species as a categorical fixed effect, and larval density (number of surviving larvae in the stack) as a covariate. Significance was assessed by chi‐square tests, and a post hoc test was conducted to determine which populations had a mean position significantly below zero. Direct‐development treatments were analysed separately. We also considered an alternative modelling strategy where the dependent variable was the proportion of larvae found below a certain height (−2, −1 or 0). As results were qualitatively the same, we only present results for the average height.

Between‐stack variation of mean vertical position was compared to the null model in which all individuals are independently drawn from the same vertical distribution. We simulated 10,000 artificial data sets with exactly the same number of replicates and individuals per stack, but in which the position of each individual was drawn randomly from the multinomial distribution observed at species level. We thus generated the expected distribution of between‐stack variation, measured as the standard deviation of mean position among stacks. We then located the observed value in the null distribution and calculated the corresponding *p*‐value. This was done for final positions only and for each population separately. All statistical analyses were conducted using the software R version 3.2.0 (R Development Core Team, 2015).

## 3 RESULTS

### 3.1 Genetic differentiation

G2 populations, following the experiment, were clearly genetically differentiated at the *OnT2* microsatellite locus (Supporting Information; Fig. S1). *F*_ST_ values between populations from different species fell in the range (0.2–1), and those within the same species in the range (0.06–0.7). *F*_**ST**_ values between our populations and geographically close populations genotyped at the same locus in earlier studies (Bourguet et al., 2014) were small: all but one (0.323) were in the range (0.019–0.145). With no exception, allelic profiles were as expected from earlier studies, exhibiting the same dominant alleles, although with reduced diversity, genetic drift presumably causing some rare alleles to be lost during the two generations of laboratory‐rearing.

### 3.2 Geotactic behaviours

In diapause‐inducing conditions, after 12 weeks, an average of 17.9 individuals—of the 40 initially introduced, *that is* 44%—had survived per stack. As expected, only a small proportion of larvae developed into pupae, albeit with some variation among populations (Fig. S2).

**Figure 2:**
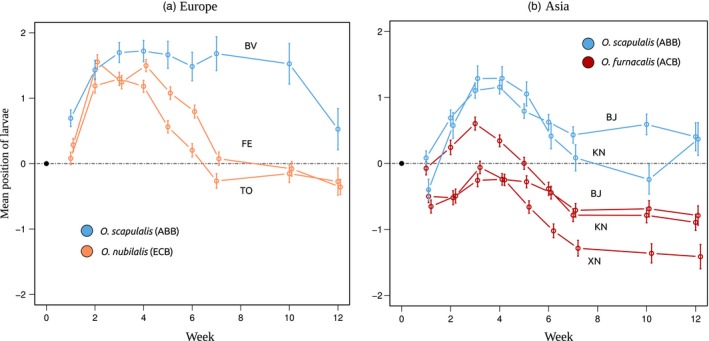
Mean vertical position of individuals in the artificial stacks over the course of the experiment (12 weeks under diapause‐inducing conditions), for European (a) and Asian (b) populations. Each population is plotted individually, and error bars represent standard errors (SE). Initially all individuals were introduced at position 0 (horizontal dashed line)

The overall trajectories of larvae in the stacks, over the duration of the experiment, are represented in Figure 2. European populations initially tended to move up (Figure 2a), indicating negative geotaxis, consistent with Calcagno et al.'s (2010) findings. The same trend was observed in Asian populations, although it was somewhat weaker for ACB (Figure 2b). Comparing European populations, ABB and ECB began to have different vertical positions on week five (Figure 2a; GLM, *p* < .01). While ABB populations retained a high mean position, staying in the upper half of the stacks, the maize‐feeding ECB went down, and eventually adopted a mean position below the initial central position. Within Asian populations, the same difference was observed between ABB and the maize‐feeding ACB. While the mean vertical position of ABB populations only slowly declined from week four onwards, converging to the initial central position, ACB populations presented a clear tendency to move down between weeks four and seven, thus adopting a mean vertical position well below the initial central position, and maintaining it until the end of the experiment.

**Figure 3:**
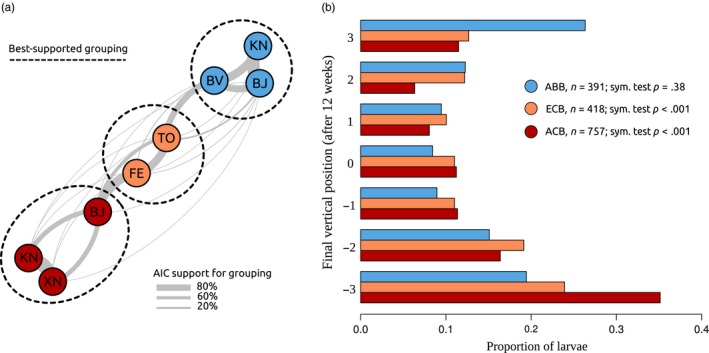
(a) Clustering analysis of the eight populations based on their final vertical distributions. Each population is represented as a node, and the thickness of edges between nodes is proportional to the relative support for a grouping of the two populations in the same cluster (legend in insert). Dotted curves represent the best‐supported population grouping, which consists in three clusters that perfectly match species. (b) Final vertical distribution of the three species, after 12 weeks under diapause‐inducing conditions

The final vertical distribution of individuals is of prime importance, as maize harvesting generally occurs at the latest stage of larval development, when larvae are entering diapause (Calcagno et al., 2010). Clustering analysis on the full final distributions revealed an optimal clustering with three groups, corresponding exactly to the three species (Figure 3a; see also Table S1). Species membership could thus be retrieved using position information only. The vertical distributions of the three species are shown in Figure 3b (see also Fig. S3). ABB adopted a final vertical distribution that was U‐shaped and symmetric around the central vial (symmetry test; *p *= .38). In contrast, both ACB and ECB adopted final vertical distributions that were strongly skewed downwards (symmetry test; *p* < .001 in both cases), indicative of positive geotaxis.

**Figure 4:**
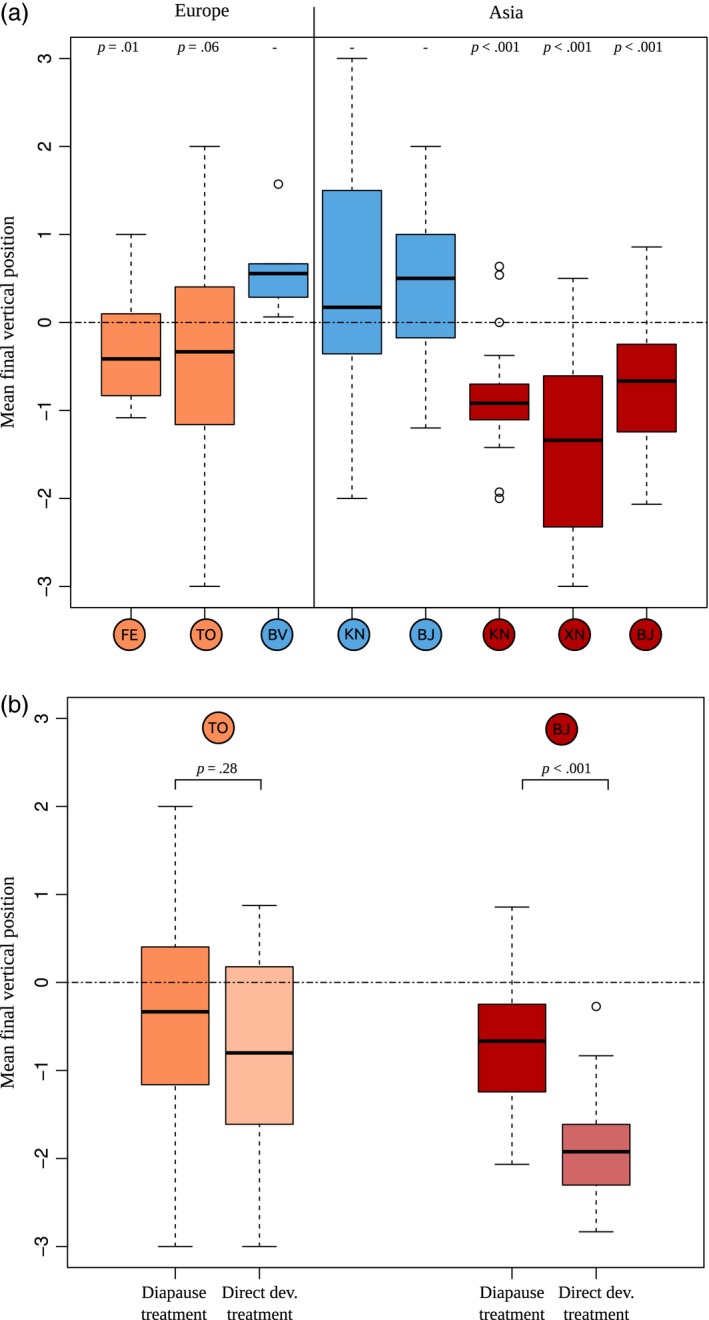
(a) Distribution of the mean final vertical positions of individuals in diapause‐inducing conditions, over replicates (stacks), for the eight populations. (b) Distribution of the mean final vertical positions of individuals in diapause‐inducing versus direct‐development conditions, for the TO ECB and the BJ ACB populations

A more specific analysis of the mean final position of larvae in stacks yielded similar conclusions (Figure 4a). The full model incorporating population as a fixed effect was not significantly better than the simpler model taking only species into account (Likelihood Ratio Test; *p *= .11). No ABB population had a mean position lower than zero (the initial central position), whereas all ACB and ECB populations did have a mean position lower than zero. Note that the test was only marginally significant for the TO ECB population (Tukey's HSD test; *p *= .06; Figure 4a). This likely reflects lack of power, as this southern population experienced a higher‐than‐average rate of pupation, which reduced the final sample size for this analysis (Fig. S2).

Mean vertical position was not significantly affected by the number of individuals in the stack (chi‐square test; *p *= .19). Within populations, the distribution of mean position across stacks was almost perfectly Gaussian (Figure 4). This is an indication that individuals distributed themselves reasonably independently in the stacks. Indeed, our variable “mean position” is the average of about 17 individual variables taking integer values (the position of each individual), and the central limit theorem states that such a variable should converge to a Gaussian distribution provided the individual variables are independent. Furthermore, the observed variance of mean position among stacks was not different from that expected based on independent multinomial sampling from the aggregate distributions shown in Figure 3b (Monte‐Carlo test: *p *= .97, .84 and .91, for ABB, ECB and ACB, respectively; see also Fig. S4). Overall, everything indicates that *Ostrinia* individuals from each species have a certain geotactic behaviour robustly described by the vertical probability distributions shown in Figure 3b, without individual interactions, density dependence or stack variation playing any important role.

### 3.3 Role of developmental pathway and environmental conditions

In diapause‐inducing conditions, we compared, for each species, the mean vertical position of larvae and newly pupated individuals within a stack. We found no significant difference on the final week between the mean position of larvae and the mean position of pupated individuals (paired *t*‐tests; all *p*‐values >.05). A small pupation peak occurred on week 10 (Fig. S2), but comparisons at this week were also nonsignificant.

In the direct‐development treatment (i.e., where larvae underwent temperature conditions favouring pupation rather than diapause), for the two populations assessed (TO ECB and BJ ACB), 23.1 individuals on average survived per stack. Almost all larvae developed into pupae (Fig. S5). The pupation peak was higher and more precocious, occurring around week six, than under diapause‐inducing conditions. The mean position of individuals on that week was much lower than the initial central position, zero (Figure 4b). For TO ECB, mean position was not statistically different from the final position adopted by the same population in diapause‐inducing conditions (two‐tailed *t*‐test; *t *= 1.09; *p *= .28). For BJ ACB, mean position under direct development was even lower than final mean position in diapause‐inducing conditions (two‐tailed *t*‐test; *t *= 5.04; *p* < .001).

## 4 DISCUSSION

Our results show that the two corn borer species, ACB in Asia and ECB in Europe, have independently evolved a tendency to move down (positive geotaxis) in the latest stages of their larval development. This phenotype is not observed in their sister species, the ABB, which feeds on various Dicots but not on maize. ABB populations, be they from Asia or Europe, do not exhibit any geotactic preference during late larval development and adopt vertically symmetric distributions. Even though virtually nothing is known about the geotaxis preferences of other *Ostrinia* species or the sister group *Pyrausta*, these taxa present life cycles more similar to the ABB, and nothing in their ecology would, to our knowledge, justify the evolution of a positive geotaxis behaviour. For example, *Ostrinia penitalis*, the American lotus borer, typically pupates in the upper parts of petioles or flower buds, and pupating close to the ground would be counter‐adaptive as lotus grows in the water (Ainslie & Cartwright, 1922). Similarly, *Pyrausta* moths are usually reported to pupate in protective chambers formed by curling and gluing host leaves, with no clear need to move to the ground. Studying the geotactic behaviours of further *Ostrinia* species would of course be very valuable, but it seems very unlikely that positive geotaxis is an ancestral character in this group. Our results thus provide strong support to the hypothesis that behaviour, specifically positive geotaxis of late larval stages, evolved repeatedly as an adaptation to maize during the two host‐shifts to this crop.

In both Asia and Europe, harvesting selectively kills larvae in the upper part of maize stalks. It is plausible that this harvest selection has been strong enough in modern times to cause the very fast appearance of the observed behaviour. Even before modern combined harvesting, burning maize stems was advocated as a method to manage ECB and limit infestation (Brindley & Dicke, 1963; Caffrey & Worthley, 1927). In the early stages of maize cultivation, harvesting techniques typically involved removing the upper parts of the stalks, and the latter had variable fates, being either burnt or stored to feed cattle over winter. Hence, a vertical gradient in mortality probably always existed. Survival in the upper parts of the stalks was maybe higher than now, but since overturning/slicing of maize stubs after harvest was not implemented yet, survival in the lower parts was certainly higher as well, somewhat restoring the gradient. Still, whereas no alternative selection pressure can conceivably have rivalled harvest selection in terms of intensity or ubiquity in the past 100 years, we cannot rule out that in the initial 300–400 years of maize cultivation, some unknown selection pressure also played a role. If the selective pressure still exists, one could test for that by measuring the current over‐wintering survival of corn borers along maize stalks in control (nonharvested nontreated) crops. For instance, there might be a vertical gradient in predation or parasitism rate (even though most parasitoids attack larvae in earlier stages), so that positive geotaxis might also constitute an escape from nonhuman natural enemies. To account for the observed behavioural differences, however, this would have to be true in both Asia and Europe, and the gradient should be maize‐specific.

Geotaxis behaviours showed little geographic variation within species, to the point that species number and species membership could be retrieved using only information on final vertical distributions, despite sampling locations being several hundreds or thousands kilometres apart and populations showing clear genetic differentiation within each species. In addition, the existence of positive geotaxis in the ECB but not in the ABB, as reported by Calcagno et al. (2010), could be re‐established in this study using a different and simpler experimental set‐up. These elements indicate that the phenotype is robust and essentially fixed in these species. This robustness is further supported by the fact that larval density, environmental conditions and developmental pathway (diapause or direct development) did not impact its expression importantly. Positive geotaxis appears to be tightly associated with the completion of larval development, but not specifically with the onset of diapause. Such a robust phenotype may thus be amenable to characterization at the level of individuals, allowing for easier and faster screens of intra‐ and interspecific variation. In conjunction with classic genetic techniques or transcriptomics analyses, this could reveal the molecular bases of the phenotype. To date, no ABB population in the four tested (three in this study and one in Calcagno et al., 2010) was found to express the geotactic behaviour. Clearly, interesting perspectives revolve around the question of the genetic basis of this phenotype in ACB and ECB. Was the same determinism recruited during both host‐shifts? In addition, is the relevant genetic variation found at low frequency in ABB or is it entirely novel?

Infestation by *Ostrinia* larvae during the growing season can be controlled by insecticide treatments, the release of biological control agents and the cultivation of transgenic *Bt* maize (Nafus & Schreiner, 1991). The former two do not provide effective and consistent control (Saladini, Blandino, Reyneri, & Alma, 2008; Smith, 1996) and the cultivation of Bt crops remains controversial and has not been adopted in several countries, notably in the European Union. In all cases, controlling the overwintering larvae that survived harvesting is particularly important, as a survival rate as low as 1.3% may suffice to maintain a threatening corn borer population (Hudon et al., 1989). These larvae can be physically destroyed, for instance by burning. Vinal and Caffrey (1919) reported that during an ECB outbreak in 1898 in Hungary, it was recommended to uproot and burn infested plants after harvest. Although probably very effective, this method requires considerable labour and expense and is rarely, if ever, employed. Tillage and mowing are two other effective means of killing overwintering larvae. For instance, it was shown that mowing down maize in the fall, followed by chisel ploughing in the spring, could cause a mortality of over 95% in overwintering larvae (Schaafsma, Meloche, & Pitblado, 1996). However, in the last decades, concerns about soil, water and energy preservation have caused a shift away from tillage (Lal, Reicosky, & Hanson, 2007 *)*. Most producers who do not plough do not mow down maize stubs either (Schaafsma et al., 1996). This decrease in ploughing and mowing might further strengthen the selection pressure favouring a positive geotactic larval behaviour in the ECB and ACB.

Our results indicate that corn borers in Asia and Europe have in a similar manner evolved a positive geotaxis behaviour that increases their survival in harvested maize crops and makes them harder to control. They further suggest that maize‐associated taxa beyond the corn borers, including pests like *Sesamia nonagrioides* or *Diatrea* stalkborers, can also be expected to evolve, or have evolved, a tendency to move down at harvest time. This could be relatively easily tested using the same methods as in the present study. The expectation might even extend to other crops that are similarly harvested by man: for instance, in sugarcane stalks, that are burnt prior to harvesting, survival of *Diatraea considerata* larvae was found to be almost 90% in underground stalks, compared to only 60% in stalk tops (Vejar‐Cota, Rodriguez‐del‐Bosque, & Caro, 2009). An intriguing possibility is that entire crop‐affiliated arthropod communities have evolved a similar harvest‐induced geotaxis syndrome to accommodate harvesting practices.

## DATA ARCHIVING STATEMENT

Data for this study are available at the Dryad Digital Repository: https://doi.org/10.5061/dryad.1pm64.
