# Supplementary material for: Parallel evolution of behaviour during independent host‐shifts following maize introduction into Asia and Europe
Source: Evol Appl. 2017 Jun 17;10(9):881–9. doi: 10.1111/eva.12481 (PMC5680425; doi:10.1111/eva.12481)
Supplement: Supplementary file 1 [file EVA-10-881-s001.pdf]

Supporting Information

Figure S1: Genetic differentiation of our eight populations at the end of the experiment. Populations are shown as nodes in a graph where edge weight is equal to one minus the pairwise F_{ST} between the two populations the edge connects. More genetically similar populations thus tend to be grouped together.

Figure S2: Fraction of individuals surviving as a larva (dark gray) and pupating (light gray) over the course of the experiment, in the diapause-inducing treatment.

Figure S3: Vertical distributions of the three species after (A) one week and (B) four weeks.

Figure S4: Variability among stacks (standard deviation) of mean vertical position. Histograms represent the expected distribution of values in simulated artificial datasets, with larvae drawn independently from the overall vertical distributions observed (shown in Figure 3b). Vertical bars represent the observed variability level.

Figure S5: Fraction of individuals surviving as a larva (dark gray) and pupating (light gray) over the course of the experiment, in the direct-development treatment.

Table S1: Best models (AIC and relative weight, on all 4,140 candidate models) for the clustering analysis of the eight *Ostrinia* populations based on their vertical distribution on week 12 in the diapause-inducing treatment. Each model is described by the number of clusters (species) it identifies, and by the assignment of each population to each cluster.

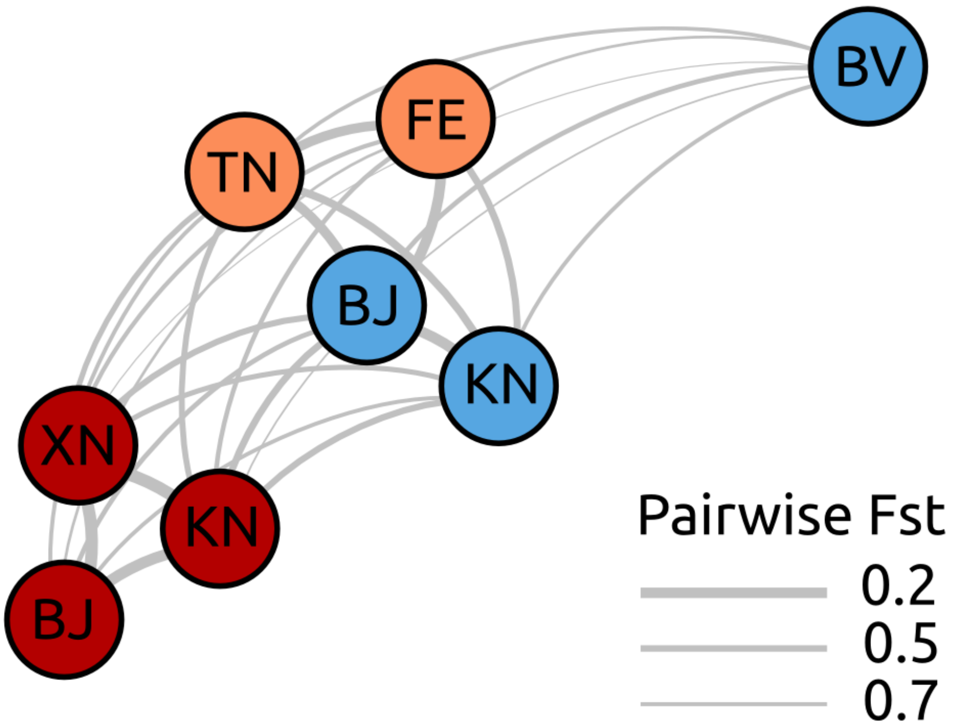

Figure S1: Genetic differentiation of our eight populations at the end of the experiment. Populations are shown as nodes in a graph where edge weight is equal to one minus the pairwise F_{ST} between the two populations the edge connects. More genetically similar populations thus tend to be grouped together.

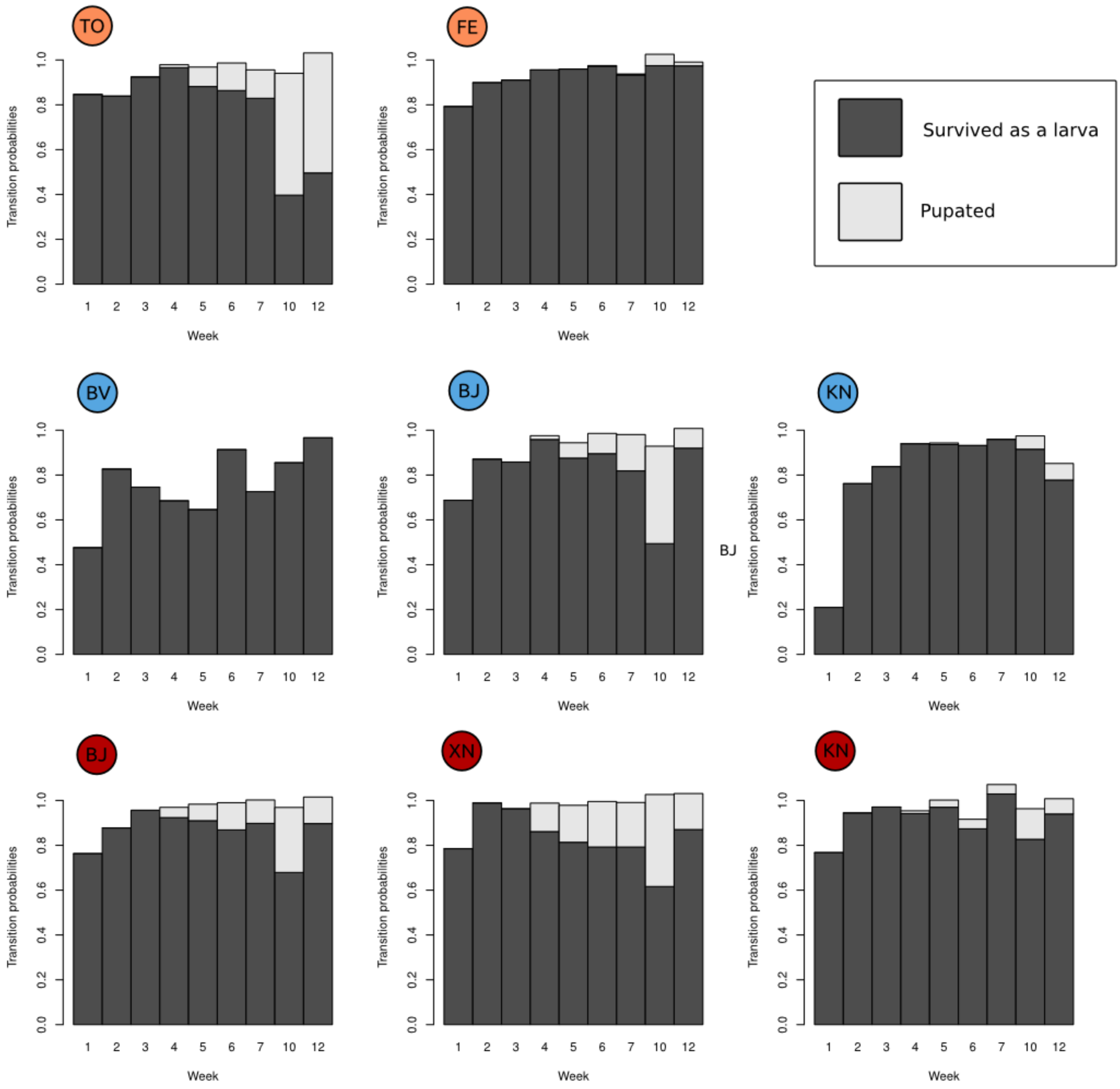

Figure S2: Fraction of individuals surviving as a larva (dark gray) and pupating (light gray) over the course of the experiment, in the diapause-inducing treatment.

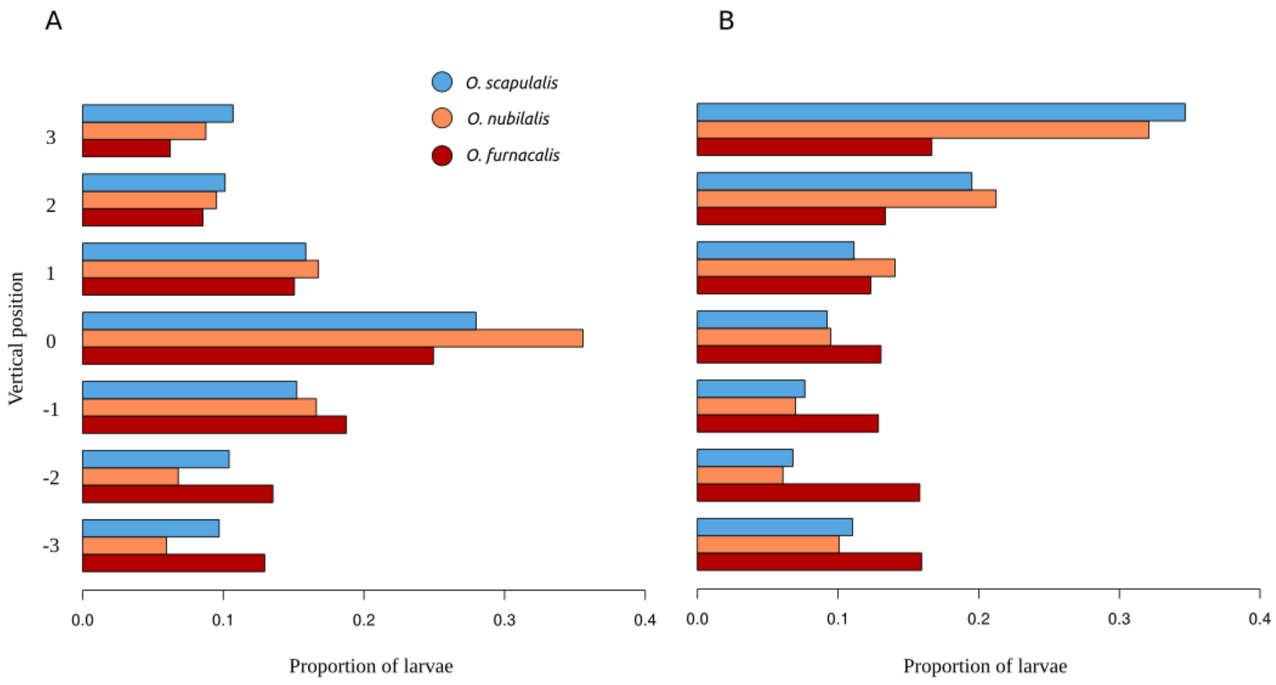

Figure S3: Vertical distributions of the three species after (A) one week and (B) four weeks.

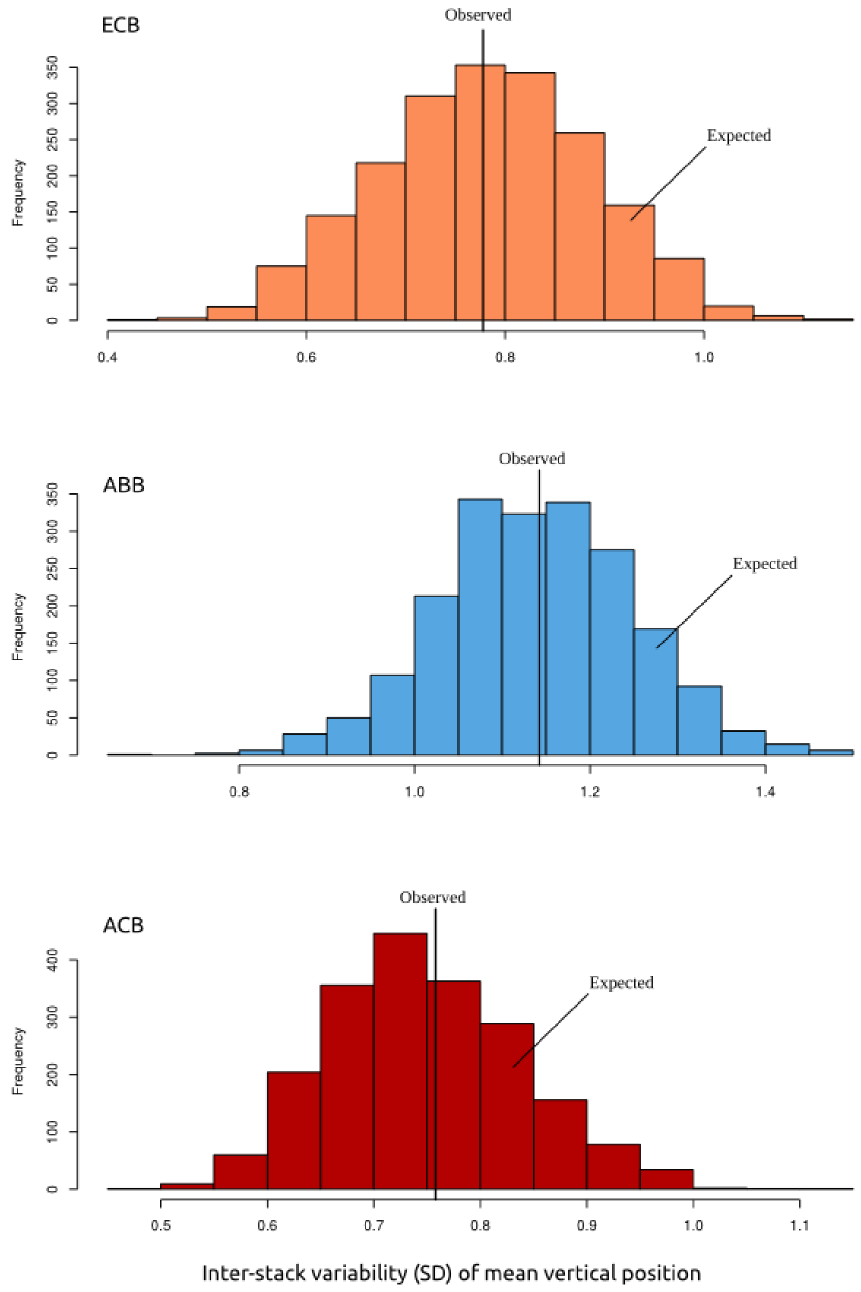

Figure S4: Variability among stacks (standard deviation) of mean vertical position. Histograms represent the expected distribution of values in simulated artificial datasets, with larvae drawn independently from the overall vertical distributions observed (shown in Figure 3B). Vertical bars represent the observed variability level.

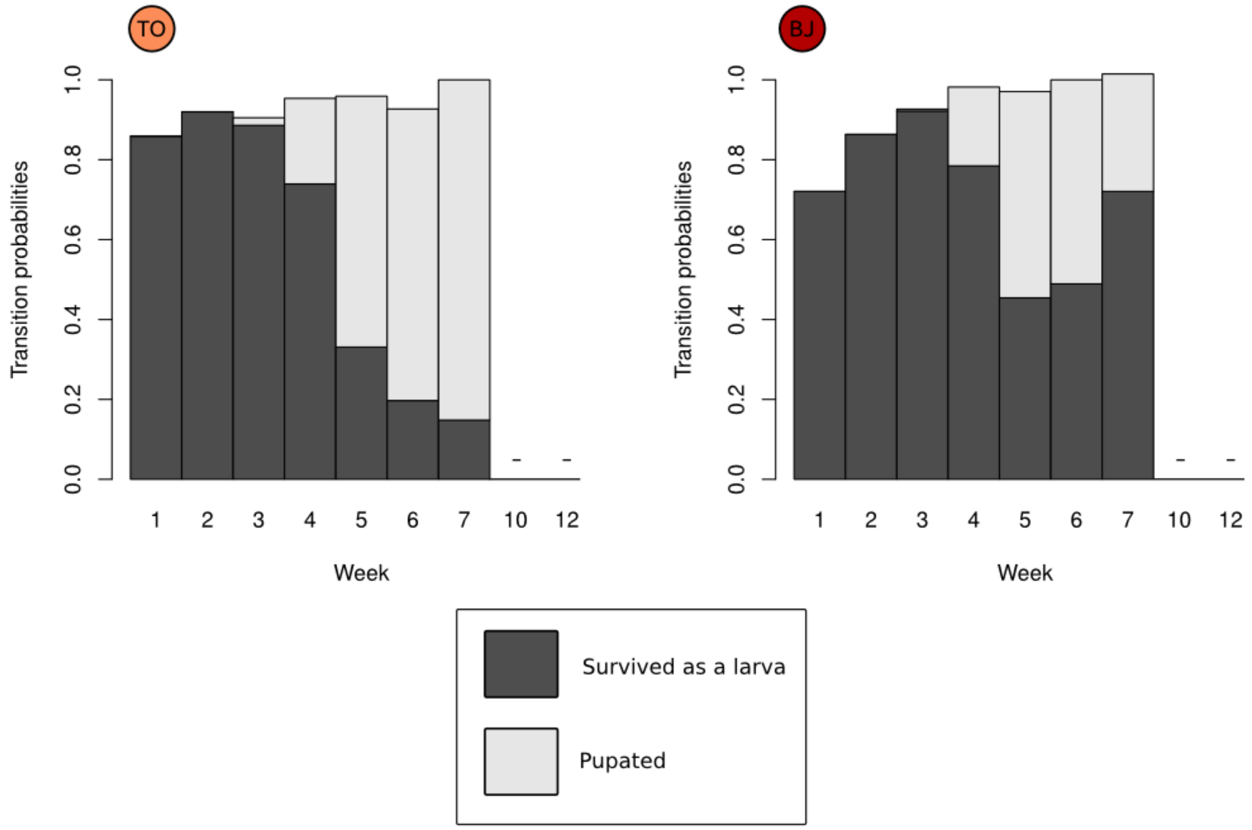

Figure S5: Fraction of individuals surviving as a larva (dark gray) and pupating (light gray) over the course of the experiment, in the direct-development treatment.

Table S1: Best models (AIC and relative weight, on all 4,140 candidate models) for the clustering analysis of the eight *Ostrinia* populations based on their vertical distribution on week 12 in the diapause-inducing treatment. Each model is described by the number of clusters (species) it identifies, and by the assignment of each population to each cluster.

Model rank	AIC	Weight	# clusters	Population assignment							
				**FE** ECB	**TO** ECB	**BV** ABB	**KN** ABB	**BJ** ABB	**KN** ACB	**XN** ACB	**BJ** ACB
**1**	**2120.396**	**1**	**3**	**3**	**3**	**2**	**2**	**2**	**1**	**1**	**1**
2	2121.062	0.717	4	3	4	4	2	2	1	1	3
3	2121.288	0.640	3	3	3	2	2	2	1	1	3
4	2124.365	0.137	3	3	3	3	2	2	1	1	1
